# Spatial Distribution of Extracellular Vesicles, Autofluorescence and CD9 Positivity Around Chondrocytes in the Superficial Layer of Articular Cartilage

**DOI:** 10.1002/jev2.70183

**Published:** 2025-10-21

**Authors:** Florian Gellhaus, Greta Ahrens, Wiebke Lückstädt, Jan‐Tobias Weitkamp, Christine Desel, Peter Behrendt, Bernd Rolauffs, Bodo Kurz

**Affiliations:** ^1^ Department of Anatomy Christian‐Albrechts‐University Kiel Germany; ^2^ Department of Oral and Maxillofacial Surgery University Medical Center Schleswig‐Holstein, Campus Kiel Kiel Germany; ^3^ Institute of Biochemistry Christian‐Albrechts‐University Kiel Germany; ^4^ Institute of Botany Christian‐Albrechts‐University Kiel Germany; ^5^ Department of Orthopaedic and Trauma Surgery University Medical Center Schleswig‐Holstein, Campus Kiel Kiel Germany; ^6^ Sportorthopaedics Varoga/Behrendt Kiel Germany; ^7^ G.E.R.N. Tissue Replacement, Regeneration & Neogenesis, Department of Orthopaedics and Trauma Surgery, Medical Center Albert‐Ludwigs‐University Freiburg Germany

**Keywords:** cartilage, CD9, extracellular vesicles, spatial distribution, tracking

## Abstract

This study investigates the distribution of extracellular vesicles (EVs) in superficial articular cartilage, hypothesizing that EVs (a) are unevenly distributed in this zonally organized tissue and (b) share a pattern similar to tissue autofluorescence. Fresh unfixed superficial cartilage from the femoropatellar groove of bovine knees was analysed using multiphoton microscopy (second harmonic generation, SHG, and multiphoton‐autofluorescence, MPAF). Transmission electron microscopy (TEM) and immunostaining (CD9, membrane marker; collagen type VI, pericellular matrix marker) were performed on fixed tissue. Cartilage‐bone cylinders were analysed in a simulated endomicroscopic setting. Additionally, EVs were isolated from synovial fluid and chondrocyte cell culture medium to demonstrate autofluorescence and staining properties. MPAF revealed a specific spatial distribution around superficial chondrocytes: lateral ring‐like accumulations inside the cell lacunae and snow cap‐like formations above cells outside the lacunae. CD9 staining was found outside the collagen type VI‐positive matrix in MPAF‐correlating locations. TEM confirmed a similar EV distribution. The endomicroscopic setting also visualized the lateral MPAF accumulations. In tissue with early osteoarthritic degeneration these patterns were not found. In conclusion, EVs/CD9 exhibit a specific spatial distribution, suggesting guided EV transport or binding within the extracellular matrix, which changes with tissue degeneration. These findings provide insight into the spatial relation between EVs and superficial cartilage architecture in health and disease and indicate a potential link between extracellular MPAF and EVs as a basis for the development of diagnostic methods and in vivo EV tracking.

## 1 Introduction

Articular cartilage is a unique tissue that provides a nearby frictionless, but load‐bearing surface and allows the distribution of mechanical forces and torques during joint movement (Eschweiler et al. 2021). The significance of that function becomes clear when articular cartilage does not fulfil its function, resulting in pain and a loss of motion in the affected joint (e.g., in osteoarthritis) (Eschweiler et al. 2021; Guilak et al. 2018; Yao et al. 2023).

A zonal architecture of cartilage is described, where chondrocytes maintain the extracellular matrix (ECM). The cell‐surrounding pericellular matrix (PCM) is largely composed of the marker molecule collagen type VI (Zhang 2015), and functions as a filter and transductor for both, biomechanical and biochemical factors (Guilak et al. 2018; Zhang 2015). The unity of cell and PCM is also referred to as ‘chondron’ (Benninghoff 1925). Surrounding the chondron, the territorial matrix (TM) consists of thicker collagen type II fibrils (Guilak et al. 2018).

The chondrocyte's matrix maintenance is influenced by the mechanical and biochemical environment (Zhang 2015; Behrendt et al. 2018; Liu et al. 2022), for example, by signal molecules, such as growth factors or cytokines, which modulate the activity of the cells (Behrendt et al. 2018; Liu et al. 2022). Recently, extracellular vesicles (EVs) and their role in cell‐to‐cell communication are gaining attention (Yáñez‐Mó et al. 2015; Miyaki and Lotz 2018). This heterogeneous group of EVs is further distinguished into different sub‐groups such as exosomes, microvesicles or apoptotic bodies (Otahal et al. 2023; Tkach and Théry 2016; Valadi et al. 2007; Mulcahy et al. 2014). Exosomes, the size‐wise smallest group, are from particular interest since they can contain different bioactive molecules, lipids as well as RNA or DNA (Yáñez‐Mó et al. 2015; Otahal et al. 2023; Valadi et al. 2007; Raposo and Stoorvogel 2013). They are phospholipid bilayered (as the other EVs) (Yang et al. 2023), with an average size between 30 and 150 nm and are actively produced by cells through fusion of exosome‐containing endosomes with the plasma membrane of the cell (Miyaki and Lotz 2018; Tkach and Théry 2016). Since discrimination of exosomes from other membrane derived EVs, such as apoptotic bodies or microvesicles with a size of >100 nm, is difficult (Kanada et al. 2015; Crescitelli et al. 2013), markers for exosomes have been identified, for example, tetraspanins, such as CD9 (Yáñez‐Mó et al. 2015; Miyaki and Lotz 2018; Tkach and Théry 2016). However, CD9 is also present in the cell membrane, some endosomes and also the other types of EVs, but is described to be a more specific protein for exosomes (Otahal et al. 2023; Tkach and Théry 2016; Kowal et al. 2016). At the target cell, bioactive EVs either fuse with the cell membrane or are taken up via endocytosis in order to release their ingredients (Mulcahy et al. 2014; Morelli et al. 2004). For chondrocytes EVs have been described to be a factor in osteoarthritis in both, protective and degenerative processes (Miyaki and Lotz 2018; Zhao and Xu 2018; Derfus et al. 1996; Rosenthal et al. 2011). So far, the spatial distribution of EVs in articular cartilage has barely been described. Some have been shown in the PCM, in the edges of the PCM as well as in the overall ECM, where EVs are described to be randomly distributed (Poole et al. 1987; Ghadially et al. 1965; Poole et al. 1984; Ali 1977; Stockwell 1975). For the tissue, however, the most superficial layer of cartilage is a key factor in the progress of osteoarthritis as it is most vulnerable for mechanical injuries and important for load distribution (Rolauffs et al. 2010; Glaser and Putz 2002). Therefore, this study aimed to investigate the detailed spatial distribution of EVs in the superficial cartilage layer, and it was hypothesized that EVs show specific patterns of distribution in relation to the cells. Additionally, we hypothesized that EVs and tissue autofluorescence are correlated to each other, based on the potential ability of EVs to create autofluorescence (Sorrells et al. 2024), and a more specific tracking of EV autofluorescence, which has been performed in breast cancer tissue (Sorrells et al. 2021, 2024). Therefore, we evaluated the cartilage tissue via non‐labelling multiphoton microscopy, which offers the opportunity to visualize cartilage´s tissue organization via second harmonic generation (SHG) and multi‐photon‐autofluorescence (MPAF) (Mansfield et al. 2007, 2019; Novakofski et al. 2014).

## 2 Methods

### 2.1 Isolation of Bovine Articular Cartilage

In eight bovine (Bos taurus) knee joints cartilage samples including all non‐calcified tissue zones were isolated from the proximal third of the medial and the lateral facet of the femoropatellar groove with a scalpel. Samples were harvested within 24 h after time of slaughter and stored in a PBS solution (PAA Laboratories, Pasching, Germany) with 100 units/mL of penicillin G and streptomycin (PAA Laboratories, Pasching, Germany) at 9°C for a maximum of 16 h until analysis. Some cartilage samples were fixed for histological analysis in 4% (w/v) paraformaldehyde (PFA; Merck, Darmstadt, Germany) and for transmission‐electron‐microscopic analysis in 3% (w/v) glutaraldehyde (Merck, Darmstadt, Germany). To ensure vitality, a live‐dead test with calcein (Sigma–Aldrich, St. Luis, MO, USA) and propidium iodide (Sigma–Aldrich, St. Luis, MO, USA) had been performed on randomly picked cartilage samples (not shown). In three knees, additional cartilage bone cylinders were drilled out and an image plane was generated parallel to the surface. The cylinders were also fixed with paraformaldehyde as described above and sections parallel to the tissue surface were generated to simulate the same view for immunostaining with collagen type VI and CD9. After sample harvesting, the knees were further dissected and inspected visually to ensure the absence of any cartilage erosion or degradation in the entire joints.

### 2.2 Isolation and Purification of EVs

EVs were isolated from two sources. (A) Synovial fluid, which was taken after opening of the knee joints and which was diluted 1:5 in PBS to reduce viscosity; and (B) from cell culture medium from isolated chondrocytes from the same joints. For chondrocyte isolation the cartilage was cut into 1 × 1 × 1 mm samples and then digested for 2 h in 0.1% pronase (Roche Diagnostics GmbH, Mannheim, Germany) and hyaluronidase (Sigma–Aldrich, St. Luis, MO, USA) solution in Alpha‐MEM Eagle (PAN Biotech GmbH, Aidenbach, Germany) at 37°C, followed by a 14 h digestion in a 600 U/mL collagenase II in Alpha‐MEM Eagle solution. Isolated cells were cultured for 3 days in a monolayer at 37°C with 5% CO_2_ in Alpha‐MEM Eagle medium including 10% of exosome free foetal bovine serum (Sigma–Aldrich, St. Luis, MO, USA), supplemented with 0.1 mM amino acids (Sigma–Aldrich, St. Luis, MO, USA), 1% ITS liquid media supplement (Discovery Labware, Inc. Bedford, MA, USA) 100 units/mL of penicillin, streptomycin and amphotericin B (PAA Laboratories, Pasching, Germany). Cell culture medium and synovial fluid were independently used for EV isolation and treated equally. Initially, cells were removed by centrifugation for 3 min at 2000 × *g* at 4°C. This was followed by a 45‐min centrifugation step at 15,000 × *g* at 4°C to eliminate all cell debris and apoptotic bodies. Subsequently, the supernatant was placed in 10 mL polycarbonat bottle 16 × 76 mm, thick‐walled, with Noryl‐cap (Laborgeraete Beranek GmbH, Nussloch, Germany) and underwent ultracentrifugation for 2 h at 100,000 × *g* at 4°C using the Optima XPN‐80 with a type 70.1 Ti fixed‐angle titanium rotor (Beckman Coulter, Brea, CA, USA). The k‐factor was calculated to 122 (38,200 rpm, rmin = 40.5 mm, rmax = 82 mm). The EV pellet was washed with 1 mL of PBS, concentrated into two tubes, and centrifugation was repeated for 1 h. The pellet was then resuspended in 200 µL of PBS and stored at 4°C for further experiments, with a maximum storage duration of 3 days.

**FIGURE 1:**
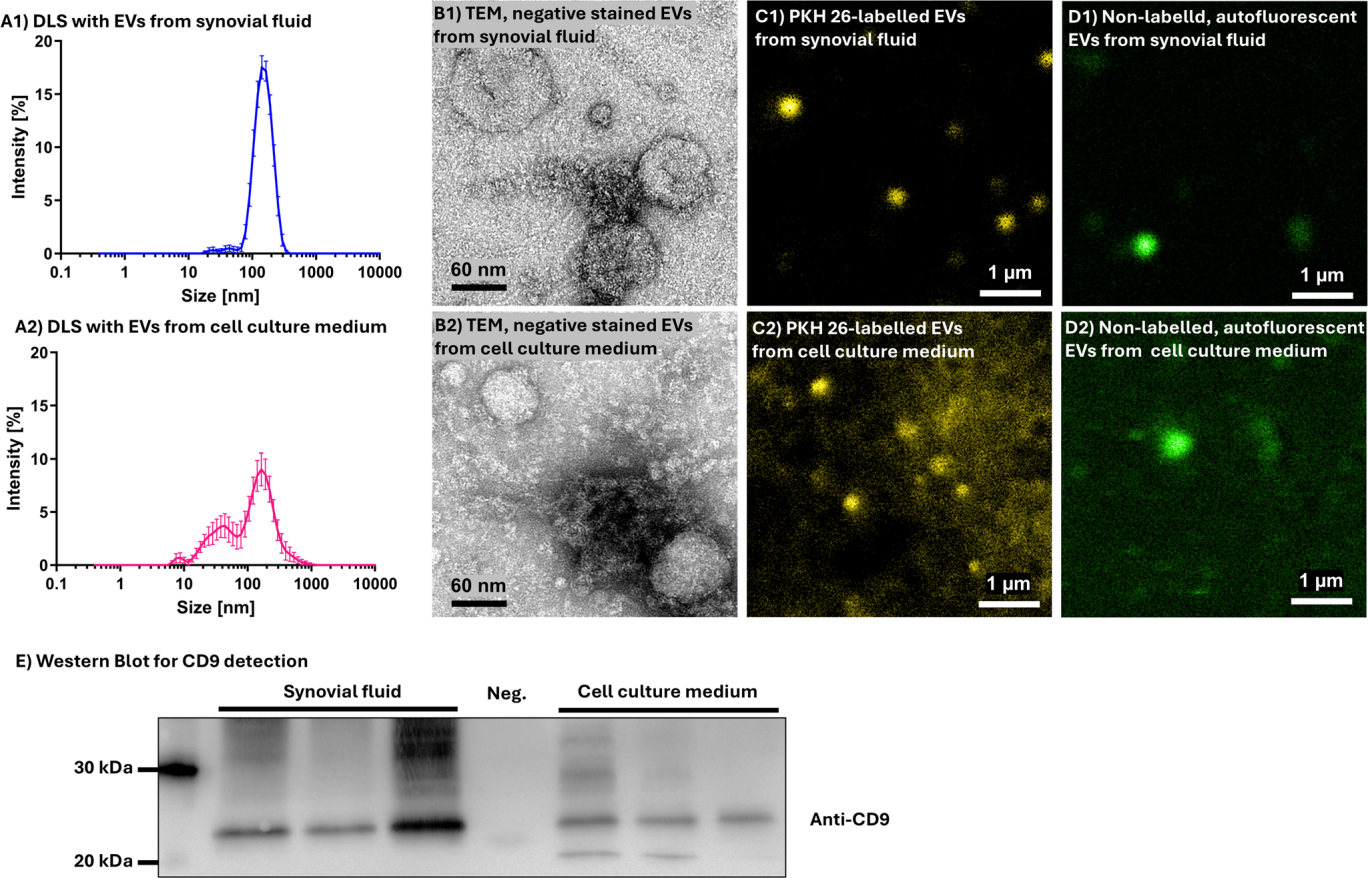
Analysis of purified extracellular vesicles (EVs) from synovial fluid (*n* = 3) and chondrocyte cell culture medium (*n* = 3). A1/A2: Dynamic light scattering (DLS) measurement of unfixed, non‐labelled EVs to determine particle size. Data displayed as mean ± standard error of the mean. B1/B2: Representative images of negative stained EVs in transmission electron microscopy (TEM). C1/C2: PKH 26‐labelled EVs in fluorescence imaging, D1/D2: autofluorescence exited in unfixed, non‐labelled EVs in a typical green channel in fluorescence imaging. E: Western blot for anti‐CD9 from in EVs generated from synovial fluid and cell culture medium, compared to the negative control (Neg.), which is the cell culture supernatant after the first ultracentrifugation. This is a representative western blot, with three independent biological replicates (*n* = 3) for synovial fluid as well as cell culture medium. Scale bar in series B: 60 nm, in series C and D: 1 µm.

For fluorescence imaging 50 µL of the EV solution were centrifugated at 100,000 × *g* for 1 h to reform a pellet. The pellet was resuspended in 50 µL Diluent C (Sigma–Aldrich, St. Luis, MO, USA). After resuspension the PKH 26 staining solution (Sigma–Aldrich, St. Luis, MO, USA) was added and the EVs stained for 5 min (final concentration 1.5 µM PKH 26). Staining was ended by adding 500 µL PBS. Again, a pellet was formed by centrifugation at 100,000 × *g* for 1 h. The pellet was resuspended in 50 µL of PBS. Samples were prepared on a coverslip for microscopy. Also, a sample of native, unlabelled EVs was generated this way. For both, a negative control was added, especially since it is well known that salts can lead to the formation of PKH 26 particles which might lead to misidentification (Figure 1A) (Haines et al. 2025). Imaging was performed using an Abberior FACILITY Line multi‐laser confocal scanning microscope equipped with a stimulated emission depletion (STED) module and a 100×, NA 1.40 objective (Olympus, Hamburg, Germany). For PKH 26 labeling, excitation was set at 561 nm, with emission detection between 571 and 681 nm, and STED depletion applied at 775 nm. Autofluorescence signals were acquired using a standard green channel with an excitation wavelength of 488 nm and detection between 498 and 551 nm.

### 2.3 Multiphoton Microscopy

After the brief storage of cartilage samples in PBS (see above) a rejuvenation cut with a scalpel blade was made to get a new cutting edge. Each explant was then placed in modelling clay (Feuchtmann GmbH, Burgbernheim, Germany) on a standard microscope slide, with the fresh cutting edge pointing upward. It was then embedded in non‐modified PBS, a coverslip (Thermo Fisher Scientific, Waltheim, MA, USA) was placed on top to avoid tissue displacement and on that coverslip a droplet of PBS was applied. The cartilage‐bone cylinders were fixed using the model clay on a standard slide and adjusted so that the image plane aligned parallel toward the surface and PBS were again used for imaging, simulating an endomicroscopy view of the tissue. The slide was then transferred to the modified, non‐inverted LSM 880 laser‐scanning‐microscope (Carl Zeiss, Oberkochen, Germany). The objective (20× water‐immersion, Plan‐Achromat, numerical aperture 1.0, n_D_ = 1.33, Carl Zeiss, Oberkochen, Germany) was lowered down into the PBS. It was ensured that no air remained in the liquid. Imaging acquisition was done with the Zen Black software (Carl Zeiss, Oberkochen, Germany) under the following settings: Excitation was performed using a Ti:Sapphire‐laser (Spectra‐Physics, Milpitas, CA, USA) with a monochromatic wavelength of 820 nm and a time per pulse of 12.5 fs. With a dichroitic mirror the backscatter signal was forwarded into a BiG detector (Carl Zeiss, Oberkochen, Germany), splitted there with a dichroitic mirror (350–458 nm reflection, transmission 464–900 nm) for parallel imaging of SHG (390 ± 40 nm) and MPAF (525 ± 50 nm) signals (all AHF, Tübingen, Germany).

### 2.4 Classical Histological Analysis and Immuno‐Histological Staining

Cartilage samples were fixed overnight using 4% paraformaldehyde in PBS, embedded in Paraplast (Carl Roth, Karlsruhe, Germany) and 5 µm‐thin serial sections were cut sagittal through the entire thickness of the explants and immobilized on glass slides. Sections were stained with toluidine blue (Sigma–Aldrich, St. Luis, MO, USA) and graded and staged by two investigators with the grading system of the Osteoarthritis Research Society International (OARSI grading) and scoring system according to Pritzker et al. (Pritzker et al. 2006) and Little et al. (Little et al. 2010). Also, another set of sections was co‐immunostained for both, Cluster of differentiation 9 (CD9) (monoclonal mouse IgG; 1:300 dilution, #MA1‐19301 Invitrogen, Thermo Fisher Scientific, Waltheim, MA, USA) and collagen type VI (rabbit polyclonal IgG; 1:300 dilution; #ab6588, abcam, Cambridge, UK). Cell nuclei were stained using bisBenzimid H 3334 (Merck, Darmstadt, Germany). Sections parallel to the surface were treated the same way as described above and stained using the same protocol. Absence of autofluorescence during image acquisition was ensured due to low exiting laser intensity and exposure time. Cryo‐sections were made by snap‐freezing cartilage samples embedded in cryo‐matrix (Thermo Fisher Scientific, Waltheim, MA, USA) immediately after harvest in liquid nitrogen. Seven micrometres sections were cut using a cryotome (2800 Frigocut E, Reichert‐Jung/Leica, Wetzlar, Germany). Staining was performed similarly to the PFA‐fixed sections, but with the primary antibody incubated at 37°C over night.

### 2.5 Transmission Electron Microscopy (TEM)

Cartilage samples were fixed overnight using 3% glutaraldehyde in PBS and embedded in Araldite M (Sigma–Aldrich, St. Luis, MO, USA). Firstly, semi‐thin sections were cut from the block and stained with toluidine blue to identify the cartilage surface in the araldite sample block. After identification the block was further trimmed down in size and then 45 nm serial sections were cut sagittally through the superficial zone of the cartilage and immobilized on copper grids (Electron microscopy sciences, Hatfield, PA, USA). For contrasting uranyl‐acetate (Merck, Darmstadt, Germany) was used. Analysis was performed using a JEOL 1400 PLUS (JEOL, Akishima, Tokyo, Japan). Partly, images were spread over a 5 × 5 scheme and acquired in high resolution using the inbuild Spotscan‐function of the EM‐MENU (TVIPS GmbH, Gilching, Germany) and stitched together manually using CorelDRAW 2019 (Corel GmbH, Munich, Germany). The same software was used to identify and label extracellular vesicles manually in high resolution.

Negative staining of isolated EVs for TEM was performed as described before (Lückstädt et al. 2021). Negative staining samples were prepared utilizing glow‐discharged carbon‐coated electron microscopy (EM) grids (Electron Microscopy Sciences, Hatfield, United States). The glow discharge was conducted using the Mini Sputter Coater System (Quorum Technologies, Lewes, United Kingdom) with a current of 25 mA for 30 s. The EV sample was applied to the glow‐discharged carbon‐coated EM grid for 30 s, after which excess volume was removed using filter paper. The EM grid was subsequently stained twice with a 1% aqueous uranyl acetate solution (Merck Millipore, Billerica, MA, United States), blotted again with filter paper, and allowed to air dry. Micrographs were acquired using a JEOL 1400 Plus TEM (JEOL Germany, Munich, Germany) operating at 100 kV with a nominal magnification of 120,000.

### 2.6 Dynamic Light Scattering (DLS)

For DLS measurements, extracellular vesicle (EV) samples were prepared through a centrifugation process (5 min at 15,000 × *g* at 4°C) to eliminate large particles that could interfere with the measurements. A volume of 100 µL of the EV sample was carefully introduced, ensuring the absence of air bubbles, into UV‐cuvettes (ZEN0040, BRAND GMBH + CO KG, Wertheim, Germany), which were afterward placed in the Malvern Zetasizer Nano S (ZEN1600) Particle Analyzer (Malvern Panalytical GmbH, Kassel, Germany). The measurements were conducted at a constant room temperature of 25°C, with calibration performed for 5 min prior to measurement, under the control of Malvern Dispersion Technology Software (Version 5.10, Malvern Instruments Ltd., Worcestershire, United Kingdom). All samples were analysed in duplicate, and the standard error of the mean was calculated.

### 2.7 SDS‐PAGE and Western Blot

Gel electrophoresis and western blotting were performed as described previously (Lückstädt et al. 2021). Briefly, 10% acrylamide gel was freshly prepared, and 30 µL of EV samples and negative control (cell culture medium post ultracentrifugation) were mixed with sodium dodecyl sulphate (SDS)‐containing loading buffer, and proteins were denatured for 10 min at 98°C. After SDS‐PAGE, the gel was transferred to a nitrocellulose 0.2‐mm membrane (GE Healthcare, Boston, MA, United States), blocked with 3% milk in Tris‐buffered saline (TBS; 100 mM Tris–HCl and 685 mM NaCl, pH 7.5) for 2 h and then incubated with the primary antibody CD9 mAb (IVA50) (# MA1‐19301, Thermo Fisher Scientific, Waltheim, MA, USA) at a 1:1000 dilution overnight at 4°C. Afterward, the membrane was washed and incubated with an appropriate secondary antibody at a 1:10,000 dilution. Membranes have been washed again and developed using Vilber Lourmat Peqlab FUSION SL Gel Chemiluminescence Documentation System (PEQLAB Biotechnologie GmbH, Erlangen, Germany). Subsequently, whole protein staining was performed using Ponceau S staining solution (Thermo Fisher Scientific, Waltheim, MA, USA) (Supplement Figure 1B). For chemiluminescence markers, the MagicMark XP Western Protein Standard and the PageRuler Prestained Protein Ladder (both Thermo Fisher Scientific, Waltheim, MA, USA) were utilized in accordance with the manufacturer's instructions.

### 2.8 Data Analysis and Statistics

Multiphoton microscopical signals in the images were converted into grey values in Fiji (Schindelin et al. 2012) in order to objectify signal distribution. All data were analysed by using Prism 10 (Graph Pad Software Inc., San Diego, CA, USA). A D'Agostino–Pearson test was done to test for Gaussian‐distribution on every set of data. Depending on the result, a *t*‐ or Mann–Whitney‐*U*‐test was used. A *p* value < 0.05 was considered as statistically significant. Values in the text are given as mean ± standard deviation, if not stated otherwise.

## 3 Results

### 3.1 Analysis of Isolated EVs

In order to test that articular chondrocytes produce autofluorescent EVs, EVs were isolated from both, fresh synovial fluid and the cell culture medium of isolated chondrocytes 3 days after initial cell adhesion (*n* = 3 for each) using ultracentrifugation. Particle size was analysed by DLS, revealing that the majority of detected EVs had a size between 50 and 200 nm (Figure 1). The presence and structure of EVs was shown by TEM. For fluorescence visualization, isolated EVs were labelled with PKH 26, an unspecific cell‐membrane marker and fluorescence imaging conditions were optimized accordingly. To test the potential of EVs to inherent autofluorescence, unfixed and non‐labelled EVs were detected using a standard green fluorescence channel. To identify protein markers of the found EVs, CD9 was immunoblotted and found in all analysed EVs samples with a molecular weight of around 20–25 kDa (*n* = 3; Figure 1E).

### 3.2 Multiphoton Microscopy

**FIGURE 2:**
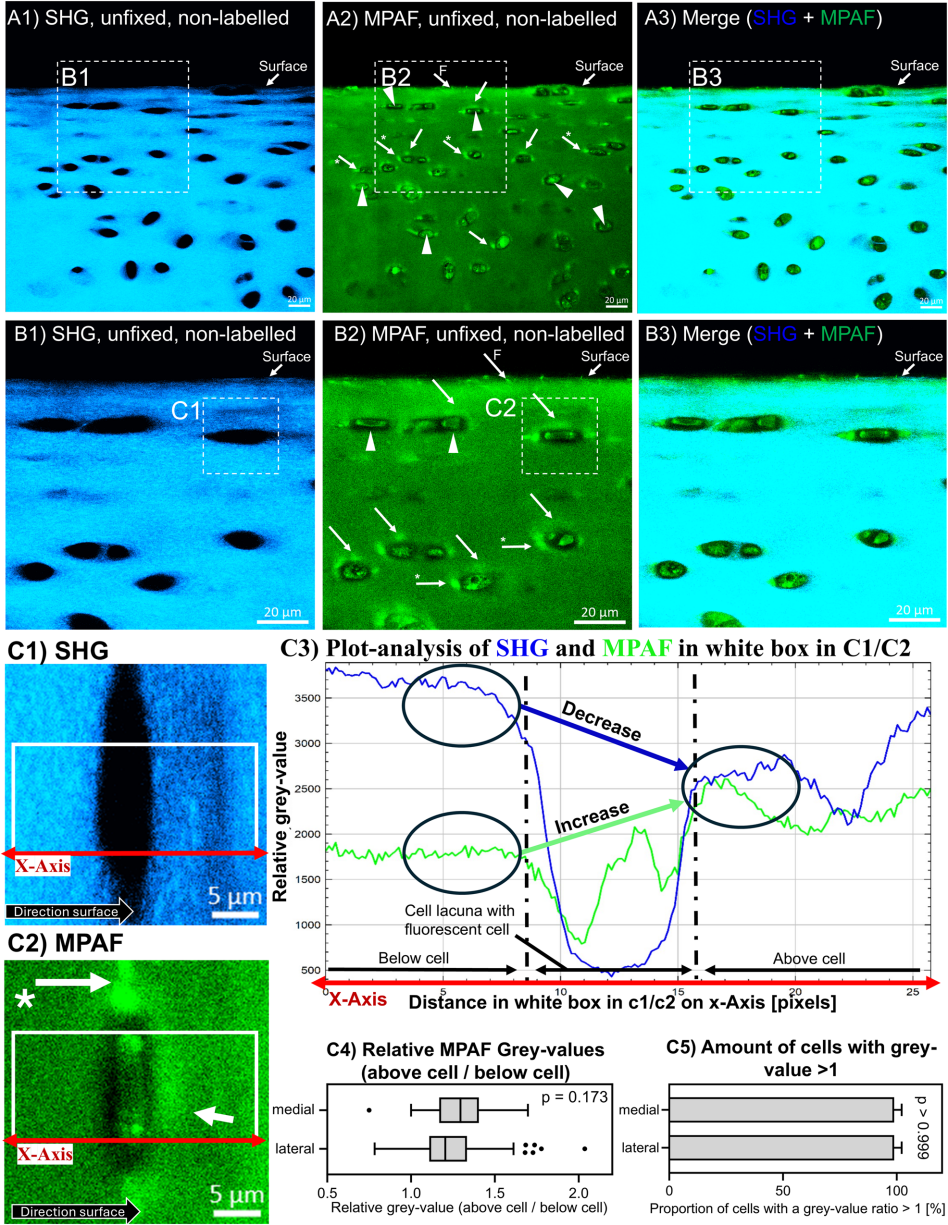
Second‐Harmonic Generation (SHG, blue signals) and Multiphoton‐Autofluorescence (MPAF, green signals) microscopy images of freshly cut unfixed, non‐labelled cartilage tissue from proximal regions of the femoropatellar groove demonstrate collagen fibrils visualized via SHG (A1, B1, C1) with typical arrangement for the superficial zone (orientation mostly parallel to surface) and cell lacunae without signals. Inside the cell lacunae, fluorescent cell‐bodies are located (triangles) with a non‐fluorescent PCM. MPAF signal intensities are increased above the cells (‘snow caps’; arrows) outside the lacunae, and lateral to the cells inside the lacunae (arrows with *). Some fibre‐like signals are found close to the surface (arrows with an F). C3: Exemplary plot analysis of the image area marked by a white box in C1 and C2 (cell taken from series B, rotated 90° clockwise). The plot is read from left to right along the x‐axis: below the cell lacuna, MPAF grayscale values follow the SHG plot; in the cell lacuna, there is no SHG, only a fluorescent cell body; above the cell, there is an accumulation of MPAF that does not correspond with the SHG signal. C4 and C5: relative ratios of MPAF accumulations calculated for values above the cell compared to those below the cell; a value >1 represents an occurrence of a ‘snow cap’. Relative values are taken in three knees with statistical comparison of the medial and lateral facet of the groove. C4: Box–Whisker plot according to Tukey (Box: upper/lower quartile, line: median, whiskers: 1.5× interquartile range, points: outliers). C5: Percentage of cells with higher fluorescence above the cell than below (= ‘snow cap effect’). Bar height represents mean + standard deviation. *p* value from direct *t*‐ or Mann–Whitney‐*U*‐test given in the Figure. Scale bars series A, B: 20 µm; C: 5 µm.

Images taken through the cutting edge of fresh native cartilage samples were done to simulate classical histological sections and the microscopical focus was deep enough inside the tissue to acquire images without any potential artefacts due to the cutting process. SHG revealed strong signals from collagen fibrils throughout the interterritorial and TM, but almost no signals in the areas of cellular lacunae (Figure 2). The orientation of collagen fibrils was mostly parallel to the tissue surface. MPAF revealed specific signal accumulations close to the cells that did not correspond with SHG signals (Figure 2, series A, B and C). These included signal intensities lateral to the cells (Figure 2, arrows with *) which were located within the SHG‐negative cell lacunae. From the lateral MPAF signal accumulations, in some cases autofluorescent stripes were rising toward the surface (Figure 4A, arrows with **). Another regular MPAF accumulation was found above the superficial cells, appearing as ‘snow caps’ (Figure 2, arrows). These signals above the cells were found outside the cell lacunae and inside the collagenous SHG‐positive matrix (the latter tended to show lower SHG signals at the same time). Additionally, MPAF showed some fluorescent fibres, mostly close to the surface, that did not produce a SHG signal and appeared as dots when cross‐sectioned (Figure 2, A2, B2, arrow with F). These were identified as elastin fibres by immunostaining (data shown in Figure S2).

The MPAF ‘snow caps’ had been quantified using a plot analysis of signal intensities in corresponding areas of the images (see examples in Figure 2, C3). When calculating a relative ratio, that is, dividing the relative grayscale value of the MPAF above a cell by the value below the same cell, a value was obtained that relatively reflects these snow cap‐like signal accumulations. A value >1 indicated an increased grayscale value above the cell compared to below. Since ‘snow caps’ were not always easily visible during live microscopy or in images, the relative ratio was determined in three knees from the medial and lateral proximal groove, 30 times in at least three different images of randomly selected superficial cells, and then statistically analysed (Figure 2, C4 and 5). Ratios within individual images were calculated instead of evaluation of absolute greyscale values to exclude errors from varying exposure times during image taking (which varied depending on the depth of microscopy within the tissue or the excitation of light intensity). The relative grayscale ratio (above vs. below) was regularly >1, revealing a stronger MPAF signal above the cells and there was no significant difference between tissue from the medial and lateral groove: in the medial facet the ratio was 1.3 ± 0.19, laterally it was 1.25 ± 0.2 (*p* = 0.173). Additionally, the percentage of cells with grayscale value greater above the cell than below (ratio > 1) was determined: 98.67 ± 3.52% of the superficial cells showed ‘snow cap’‐like MPAF signal distribution (for both, the medial and lateral facet).

In the simulated endomicroscopic setting (surface view) cells were again visible due to their fluorescent cell membrane (Figure 5, A2, triangle) and surrounded by a ring of MPAF signals (Figure 5, A2, arrow with *) which corresponded to the lateral accumulations in the cross‐sections shown above. All of the lateral MPAF patterns were again observed inside the cell lacunae, visualized by the lack of a SHG signals in that area (Figure 5, A1).

### 3.3 Transmission Electron Microscopy (TEM) of Cartilage Sections

**FIGURE 3:**
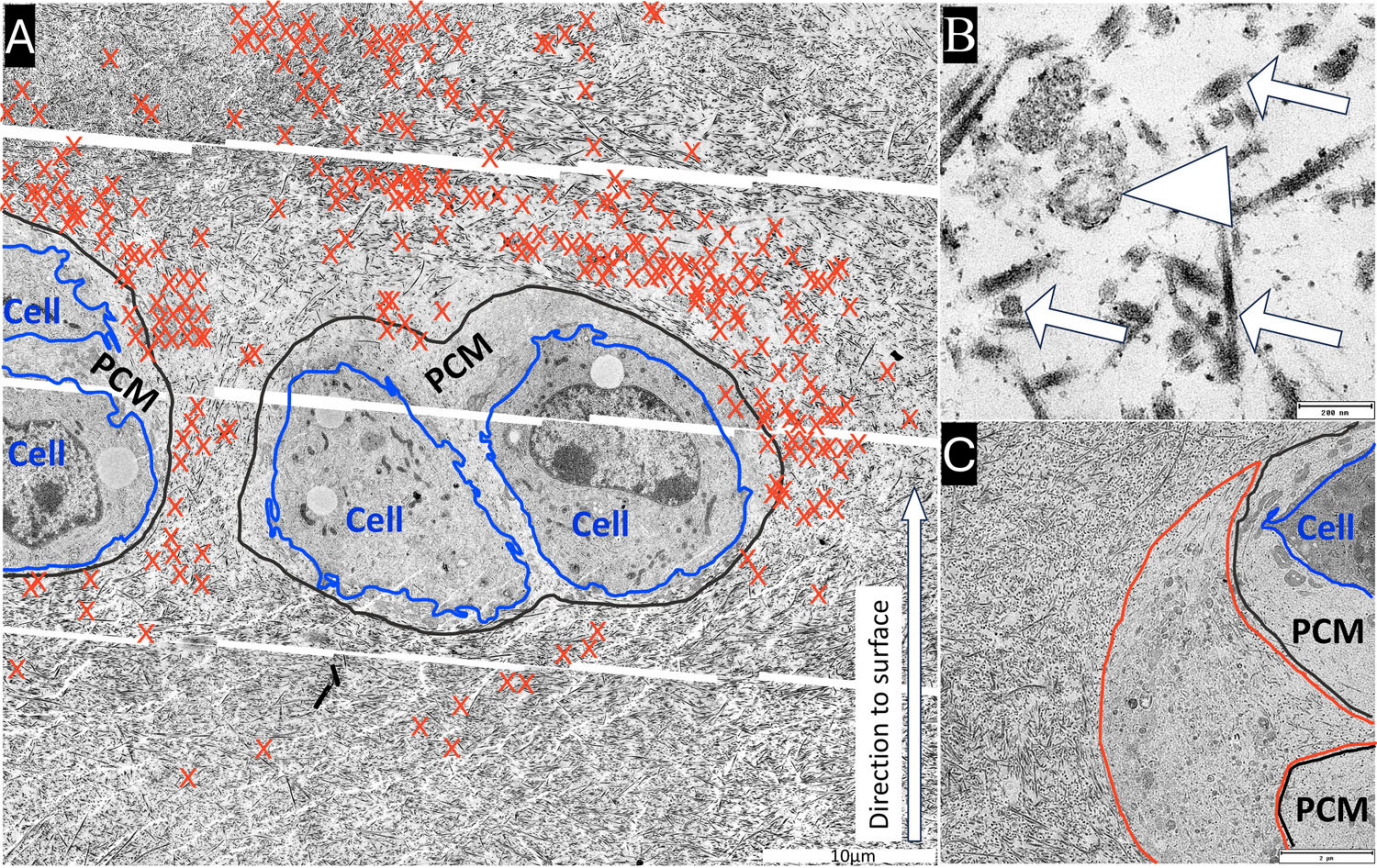
Electron microscopy images of articular cartilage tissue at the level of the most superficial cells. (A) Composite overview image with marked extracellular vesicles (EVs, red crosses), which are primarily distributed laterally and above the cells; cells and PCM highlighted with blue and black boundary lines, respectively. Only few vesicles are located below the cells. Except for the lateral location EVs are distributed among the collagen fibrils of the (inter‐) territorial matrix. (B) Triangle marks a double‐membrane vesicle in the ECM, arrows indicate collagen fibres cut transversely or longitudinally. (C) Cells (highlighted with blue outline) and a prominent accumulation of vesicles (marked by red outline) located lateral to two cells (cell below only PCM is shown) but inside the cellular lacuna which is surrounded by territorial ECM with thicker collagen fibrils. Scale bars: A: 10 µm; B: 200 nm; C: 2 µm.

For ultrastructural analysis, samples from three knees were prepared and examined. The goal was to identify EVs and their spatial distribution, which might also be the potential ultrastructural sources responsible for the observed autofluorescence patterns. Therefore, images with high resolution were stitched together to represent superficial cells and their surrounding ECM and vesicles had been identified and labelled manually with red crosses using CorelDRAW 2019 to show their location in an overview image (see example in Figure 3A).

Just like the MPAF signals described above, EVs were predominantly observed laterally to the cells inside the cell lacunae (Figure 3, A, red crosses; C, red outline) and above the cells outside the lacunae, displaying a ‘snow cap’‐like distribution, and they were rarely found below the cells (Figure 3, A, red crosses). Notably, most of these vesicles were not present within the PCM. EVs in the lateral location (related to the cells) were often densely clustered outside the PCM but inside the cell lacunae, which was indicated through the thicker collagen fibrils in the abrupt beginning TM surrounding the lacunae (Figure 3C). These lateral vesicle accumulations were especially pronounced when there was an additional cell situated above or below. The vesicles displayed considerable heterogeneity in diameters, measuring approximately 50–200 µm (as exemplified in Figure 3, B, triangle). However, there were a few outliers with diameters outside this range.

### 3.4 Immunostaining of Cluster of Differentiation 9 and Collagen Type VI

The findings described above raised the question of whether EVs might be visualized indirectly through staining of their membrane. Cluster of Differentiation 9 (CD9), a protein from the tetraspanin family, is known to be ubiquitously present in the cell membranes of mammals (and accordingly EVs). Therefore, CD9 was chosen as a marker to identify the cell membrane and potential EVs secreted by chondrocytes into the surrounding matrix.

**FIGURE 4:**
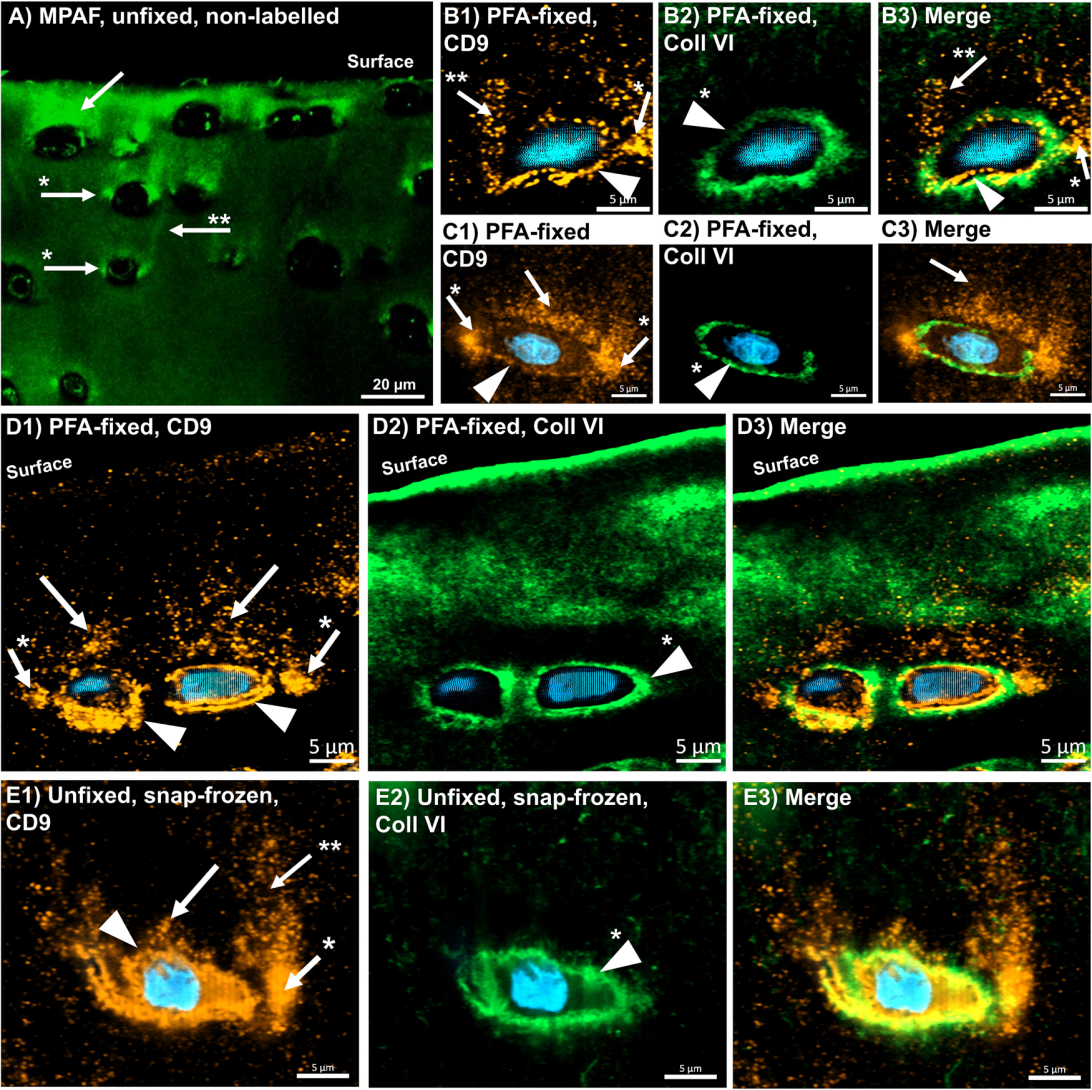
Immunohistochemical detection of CD9 (orange) and collagen type VI (green) in paraformaldehyde (PFA)‐fixed sections as well as snap‐frozen sections. A: Multiphoton‐ Autofluorescence (MPAF) image of fresh, non‐labelled cartilage for MPAF‐pattern reconnaissance. In CD9‐ and collagen type VI‐stained cartilage, the CD9 pattern matches the MPAF pattern around the cells (Series B—E). CD9 is detected at the cell membrane (triangles), lateral to the cells or cell groups (white arrows with *), with signals partially extending toward the surface (arrow with **), as well as in ‘snow cap’‐like formations above the cell groups (withe arrows). These CD9‐signals do not overlap with those of collagen type VI, which is located pericellular (triangles with *), except for those in direct contact to the cell. Nuclei are labelled by bisbenzimide (blue). To illustrate the overlap between the CD9 pattern and the MPAF pattern, identical symbols were used for corresponding signal accumulations in both types of images. Scale bars in series A: 20 µm; in series B—D: 5 µm.

The presence of CD9 in the cell membrane, particularly in association with the cell nucleus (which was counterstained by bisbenzimide), served as the internal positive control (Figure 4, triangles). Next to the cell membranes CD9 signal accumulations were consistently observed outside the PCM, as confirmed by co‐staining with collagen type VI (Figure 4, triangles for CD9, triangles with * for collagen type VI). The primary detection areas were in the territorial and interterritorial matrix above the cells (Figure 4, arrows) and lateral to the cells (Figure 4, arrows with *). In certain instances, CD9 signals were observed ascending toward the surface from the lateral regions (Figure 4, arrows with **). These lateral, laterally ascending, and ‘snow‐cap’‐like CD9 signals corresponded spatially with the MPAF distribution around superficial chondrocytes described above (Figure 4 A, same labels used as for CD9) as well as the EV distribution shown by TEM (see above). However, the localization pattern of CD9 signals was heterogeneous and not uniformly detectable around all cells. To verify that the PFA‐fixation process did not introduce alterations in CD9 distribution, CD9 and collagen type VI immunostaining were also performed on unfixed, snap‐frozen sections. These sections exhibited CD9 patterns identical to those observed in the PFA‐fixed sections (Figure 4, series E)

**FIGURE 5:**
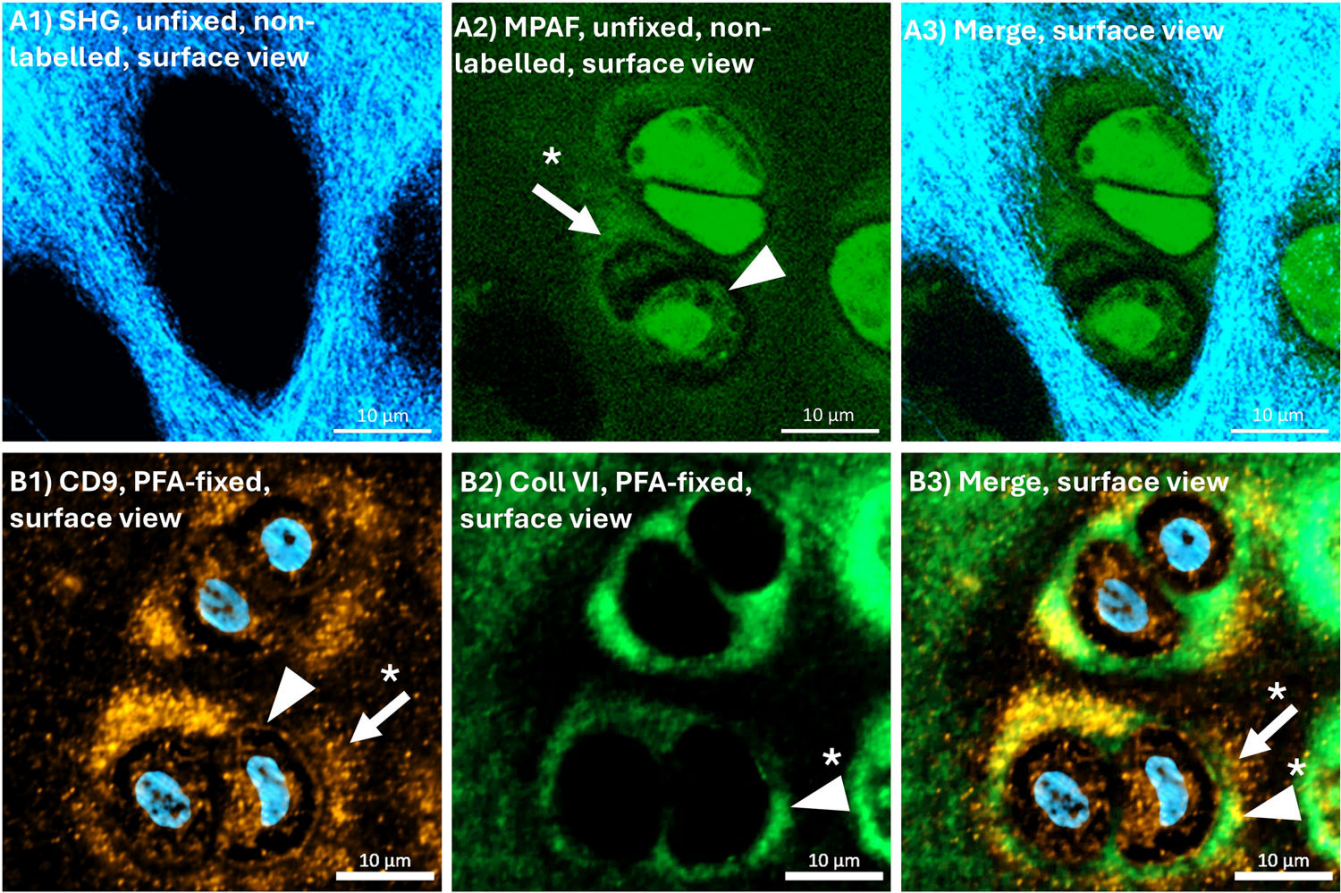
Multiphoton microscopy image of intact, unfixed, non‐labelled articular cartilage with an image plane around 10 µm below the native cartilage´s surface with a resulting image parallel to the cartilage surface (‘endoscopic view’ / ‘surface view’; series A) and immunohistochemical detection of CD9 (orange) and collagen type VI (green) in a paraformaldehyde (PFA)‐fixed histological section parallel to the cartilage´s surface to simulate a similar view (series B). The patterns of multiphoton‐autofluorescence (MPAF, A2; arrow with *) and CD9 (B1; arrow with *) do match. The main signals for both do present outside the collagen type VI‐positive pericellular matrix (PCM, B2, B3, triangle with *), partially overlapping at the edges of the PCM (potentially due to the 5 µm thickness of the section). Also, the cell membrane presents in the MPAF and is CD9‐positive (A2, B1 triangle). The CD9 accumulations outside the PCM correspond to that lateral to the cells if cross‐sectioned (Figure 4 D1‐3).

In the sections cut parallel to the native cartilages surface the cell membranes were CD9 positive as previously described (Figure 5, B1, triangle) and the MPAF signals around the cells (Figure 5, A2, arrow with *) matched with CD9‐positive accumulations (Figure 5, B1, arrow with *). The accumulations surrounding the cells corresponded to the lateral accumulations if cross‐sectioned as described above. The CD9‐positive cell membranes were surrounded by the collagen type VI‐positive PCM (Figure 5, B2, triangle with *). Partially, an overlapping in the PCM periphery was observed and, in some cases, a colocalization of the CD9 positivity and the collagen type VI‐positive PCM.

### 3.5 Toluidine Blue Staining and OARSI Grading and Scoring

Staining with toluidine blue was performed on samples of the medial and lateral facet from three of the knee joints, to ensure the absence of any early‐onset osteoarthritis. OARSI grading and scoring was done according to Pritzker et al. (Pritzker et al. 2006) and Little et al. (Little et al. 2010), respectively, by two different researchers. Proximally, in only one sample an OARSI grade of one (slight fibrillation) was observed on the medial facet, and in three samples a single score point was given, once for slight surface irregularities and twice for slightly decreased toluidine blue staining on the lateral site. It is well described, that the distal femoropatellar groove is a predisposed position for osteoarthritic lesions (Heinola et al. 2014). Therefore, tissue from the distal femoropatellar groove was also graded and staged, and tissue with OARSI grades (Pritzker et al. 2006) in between one and two was used for analysis of early degeneration‐dependent changes in the MPAF and CD9 positivity pattern around the superficial chondrocytes. Higher grades were not observed.

### 3.6 Changes in MPAF and CD9 Distribution in Early Osteoarthritic Degeneration

**FIGURE 6:**
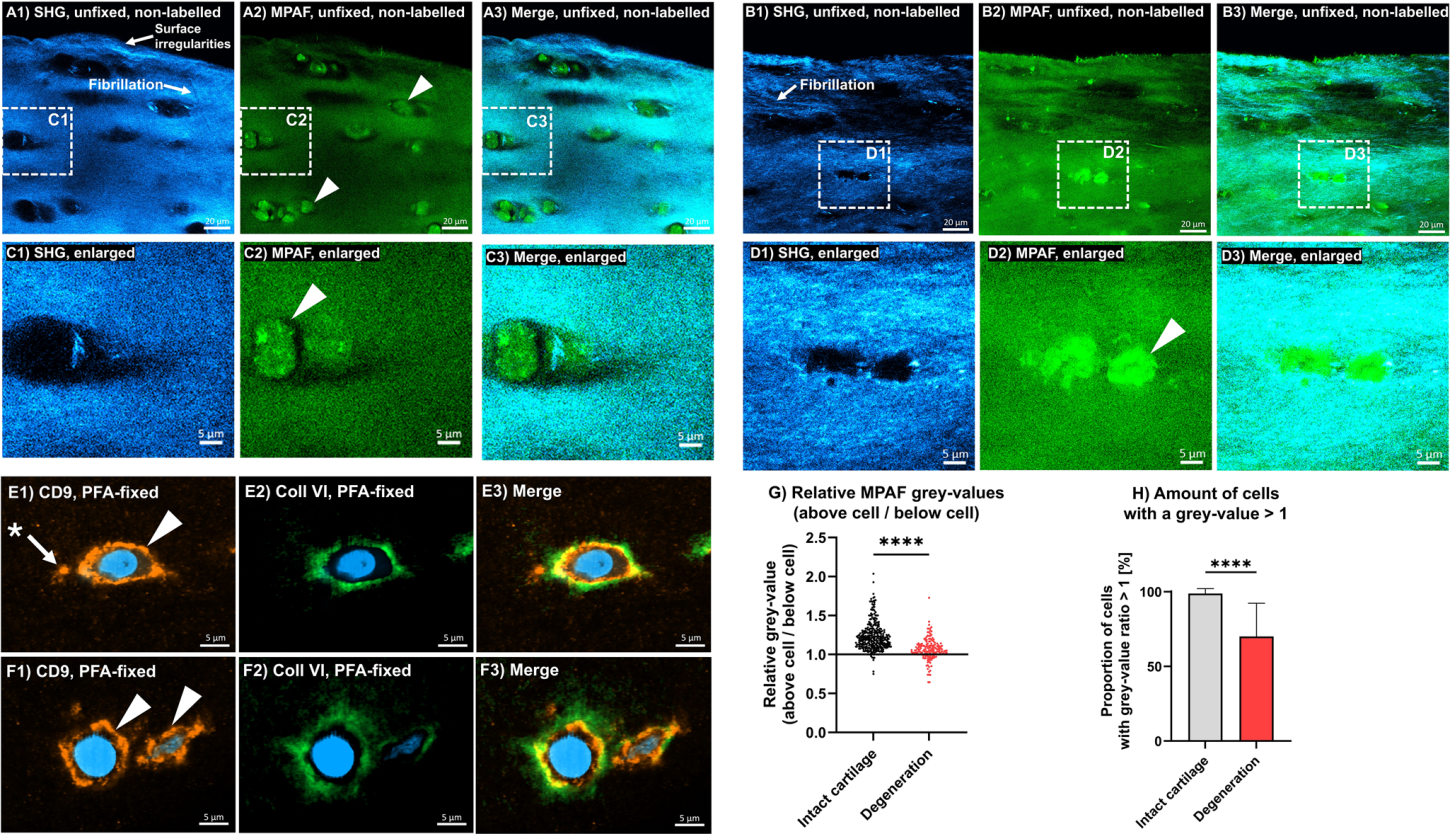
Images from Second Harmonic Generation (SHG), Multiphoton‐Autofluorescence (MPAF) and immunostaining of superficial articular cartilage with early osteoarthritic degeneration. Series A and B show collagenous fibrillation and surface irregularities in SHG imaging (arrows) known as indicators of tissue with early degeneration. Corresponding MPAF images show cells either lying inside normally defined lacunae (C2 triangle) or as strong fluorescent cells inside lacunae with irregular shape (series D, triangle). MPAF accumulations above (‘snow‐caps’) and lateral to the cells are not visible. Series E and F show immuno‐detection of CD9 (orange) and collagen type VI (green). E shows a cell with a flat appearance indicated by the CD9‐positive cell membrane (triangle) with a CD9 accumulation lateral to the cell (arrow with *) outside the collagen type VI‐positive PCM. CD9 signals above the cell are not visible. In F two cells without any CD9 accumulations outside the PCM are shown. G: Comparison of relative ratios of MPAF accumulations calculated for values above the cell compared to those below the cell; a value > 1 represents an occurrence of a ‘snow cap’. Relative values are taken in three knees with statistical comparison of the intact cartilage toward the degenerated cartilage (*n* = 3). Data given as individual dots. H: Percentage of total cells with higher fluorescence above the cell than below (= ‘snow cap effect’) in intact cartilage compared to degenerative cartilage. Bar height represents mean + standard deviation. *p* value from direct *t*‐test displayed, **** = *p* < 0.0001 (E and F). PFA: Paraformaldehyde. Scale bars series A, B: 20 µm, C–F: 5 µm.

Cartilage from the distal femoropatellar groove was analysed similarly as described above, since it is described that there might be a chance to find degenerative cartilage tissue (Heinola et al. 2014). OARSI grades in between one and two were classified as early degeneration, mainly visible as collagen fibrillation in the collagenous matrix (detectable by SHG), accompanied by surface irregularities (Figure 6, A1, B1). The cells and lacunae were no longer as flat as in healthy superficial cartilage (see above), varying from slightly to completely rounded cells. Depending on the stage of early degeneration cells were either lying inside normally formed lacunae or abnormally shaped lacunae (Figure 6, series C and D). The cell‐surrounding MAPF patterns (lateral to the cells and above the cells, ‘snow‐caps’), which had been described for healthy tissue, were less and less detectable, depending on the severity of degeneration. To objectify, again the ratio of grey value in the MPAF signal of the cells above versus the signal below the cell was calculated. With intact cartilage this ratio was 1.212 ± 0.179, in degenerated tissue 1.063 ± 0.133 (*p* < 0.0001; Figure 6G), indicating a significant reduction in the ‘snow cap’‐like distribution. The proportion of cells with a ratio >1 (‘snow‐cap effect’) was also reduced from 98.89 ± 3.23% to 70.03 ± 22.37% (*p* < 0.0001; Figure 6, H).

Immunodetection of CD9 and collagen type VI revealed that, depending on the stage of dedifferentiation, some cells still exhibited a flattened alignment parallel to the surface, as indicated by CD9 staining of the cell membrane (Figure 6, E1). In those cases, lateral CD9 accumulations outside the collagen type VI‐positive PCM were sometimes found (Figure 6, E1, arrow with *). However, this signal was reduced compared to healthy tissue. Notably, no CD9 detection above the cells (‘snow‐caps’) was found in slightly degenerated tissue. If superficial cells were formed abnormally, no CD9 positivity was found lateral and above the PCM (Figure 6, series F).

## 4 Discussion

**FIGURE 7:**
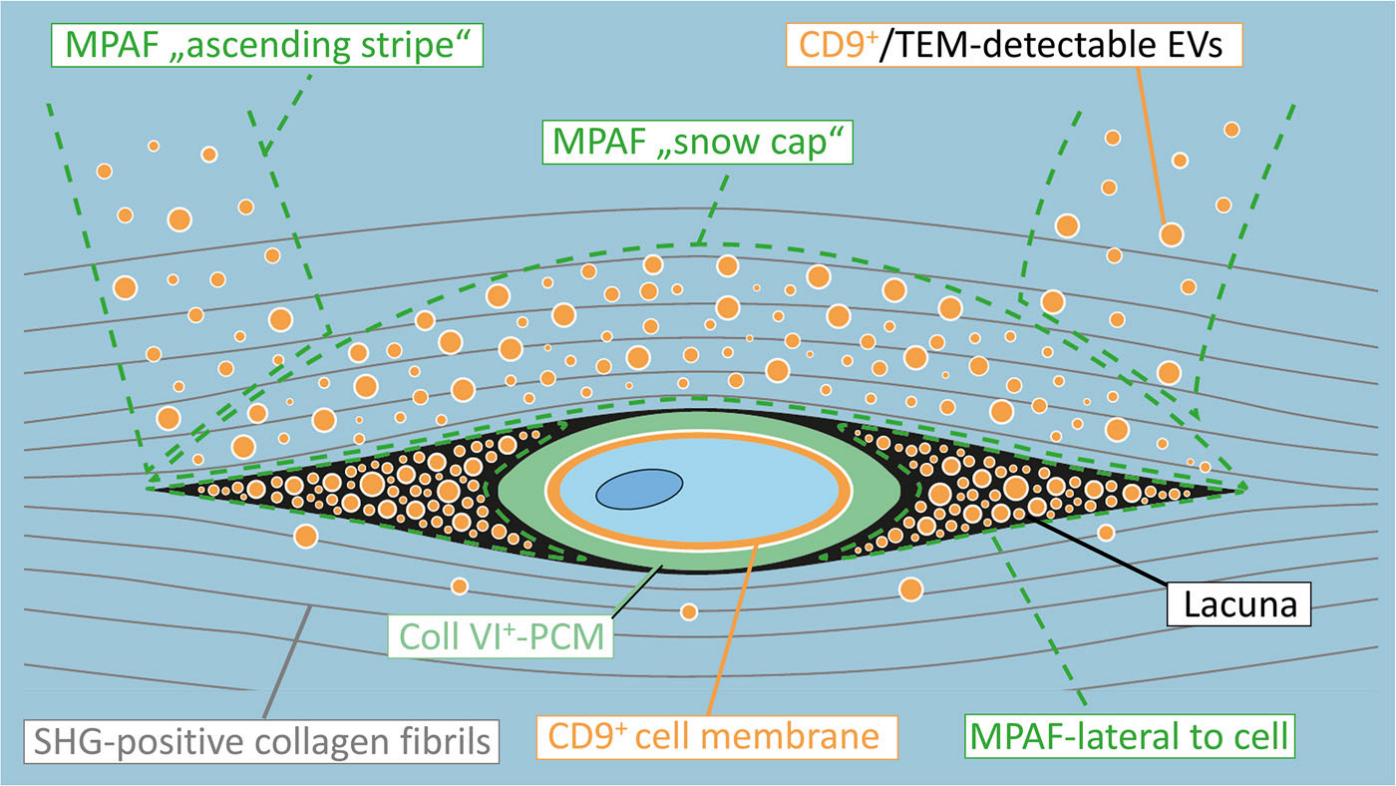
Schematic summary of the results for healthy articular cartilage. The superficial chondrocyte is surrounded by its CD9‐positive cell‐membrane (orange) and further by its collagen type VI‐positive pericellular matrix (PCM; green), all located inside a cell lacuna (black space inside the blue second‐harmonic‐generation [SHG]‐positive collagenous territorial matrix). Extracellular vesicles (EVs) are identified by CD9‐positivity and transmission electron‐microscopy (TEM). The EV accumulation patterns do match the Multiphoton‐Autofluorescence (MPAF) patterns (framed by the dashed green lines). There are three main areas of EV/MPAF‐accumulations: lateral to the cell (inside the lacuna but outside the PCM), and as ‘snow‐cap’ and ‘ascending stripes’ (inside the SHG‐positive collagenous matrix). There are very few EVs below the cell lacuna corresponding with very little MPAF signal.

The primary finding of this study is that in healthy superficial bovine cartilage EVs, CD9 and MPAF are present in very specific correlating patterns around the chondrocytes (see summarizing scheme in Figure 7). All three predominantly localize laterally to the cells with occasionally ‘ascending stripe‐like’ signals starting from the lateral regions, and additional accumulations above the cells, forming a pattern reminiscent of ‘snow‐caps.’ Notably, minimal EVs, CD9 and MPAF were detected beneath the cells. Correlating with early degenerative changes in the cartilage matrix, such as collagenous fibrillation, and degeneration‐related changes in the shapes of cells and lacunae, these patterns were reduced or missing.

The detailed spatial distribution of EVs in superficial cartilage has barely been studied previously. Different locations have been described generally: either close to the cells as well as at the lateral margins of the PCM, within the PCM or even the entire ECM (Poole et al. 1987; Ghadially et al. 1965; Poole et al. 1984; Ali 1977; Stockwell 1975). Ghadially et al. (Ghadially et al. 1965) did investigate the location of extra‐cellular lipid droplets around chondrocytes using TEM and oil red staining in human and rabbit cartilage. They already described that those droplets ‘which under the electron microscope show some membranous formations are composed partly of phospholipids’. They found them to be enriched in the most superficial layer of cartilage, especially in the first two cell layers and were described by the authors to tend to accumulate closer to cells. We also found lipid‐droplets in TEM images, but did not include them in our investigation and focused on EVs. Poole et al. (Poole et al. 1984) described the EVs to be found around the chondrocytes inside and outside the PCM of rabbit cartilage. Interestingly, they described EVs to be ‘restricted to the periphery of the pericellular matrix’ in the superficial layer of cartilage. In deeper tissue layers, they associated the location of EVs with ‘channels’ in the PCM, found above the cells. Our work demonstrates EV distribution mainly outside the PCM and PCM channels had not been observed, which is similar to human cartilage (Poole et al. 1987). The ‘snow‐cap’‐like formations found in this study for the first time are part of the territorial ECM and are not seen in the PCM. The lateral accumulations of EVs are the most described location and supposed to be at the poles or edges of the PCM (Poole et al. 1984, 1987; Ali 1977; Stockwell 1975) underlying our findings of the EV location outside the PCM, but still inside the cell lacuna, as visualized by SHG.

The ‘snow cap’‐like distribution of EVs above the cells and the ‘ascending’ stripes from the lateral accumulations have not been described previously and might be EVs being released by the cell migrating toward the cartilage´s surface and joint fluid. It is reasonable to believe this as being part of a physiological or even pathological spatial process which now needs further investigation: are the EVs fixed in that location by binding to the ECM or is this a constant process of EV transportation through the ECM toward the surface of the tissue? However, it seems that this distribution of EVs is only found in intact cartilage, because in tissue with early osteoarthritic degeneration the patterns of CD9 and MPAF around the cells were more or less missing. If cells were lying inside an intact lacuna, sometimes lateral accumulations were still observable. If the cells had lost their flat shape no specific patterns of EVs were found around or inside the lacunae. However, it remains unclear whether the loss of these patterns is due to an inability of early osteoarthritic chondrocytes to release EVs into the matrix, an altered extracellular transport mechanism (i.e., increased matrix permeability) or a disturbed binding of EVs within the matrix. However, the absence of EV patterns in early osteoarthritic superficial cartilage might be an easily detectable feature for OA diagnosis of early osteoarthritis or an important part of the OA pathogenesis.

MPAF is relatively unspecific and therefore cannot discriminate between the different components of the tissue that produce it. However, the fluorescent cells (Sorrells et al. 2024; Mansfield et al. 2009; You et al. 2019) are sharply confined (Figure 1; A2, B2) and surrounded by a non‐fluorescent ring—potentially the PCM. The CD9 – collagen type VI co‐staining revealed that the non‐fluorescent ring around the cells is indeed the collagen type VI‐positive PCM, in both, the sectioned and parallel to the surface‐orientated images. Notably, inside the lateral CD9 accumulations only minimal collagen type VI was labelled, but an overlapping was partially observed, especially at the lateral edges of the PCM. This might be an effect due to the thickness of the sections. Therefore, it is likely that these signals (and potential EVs) are found at the lateral edges of the PCM, but inside the cell lacunae. TEM supports this finding, since EV accumulations were also found lateral to the cells where the PCM was missing (Figure 2C) and inside the collagen fibrils of the territorial ECM (surrounding the lacunae), which supports previously discussed findings (Poole et al. 1987; Poole et al. 1984; Ali 1977; Stockwell 1975).

We confirmed in our study via immunoblotting that exosomes are enriched in CD9 (Figure 1), with CD positivity at around 20–25 kDa, as described for bovine CD9 (Caballero et al. 2013). The main EV size found in this study was 50–200 nm (Otahal et al. 2023; Tkach and Théry 2016; Kowal et al. 2016), Therefore, we suggest that exosomes are the main type of vesicles in superficial cartilage. However, it is a false assumption that the CD9‐positivity is found exclusively due to the presence of exosomes, since other EVs also contain CD9 (Otahal et al. 2023; Tkach and Théry 2016; Kowal et al. 2016; Théry et al. 2018; Andreu and Yáñez‐Mó 2014). A further discrimination‐method between the different types of EVs is still lacking (Otahal et al. 2023; Théry et al. 2018), and therefore, the neutral term EV is used in this study (Théry et al. 2018; Witwer and Théry 2019).

Since paraformaldehyde (PFA) fixation can induce clustering of EVs (Hobro and Smith 2017), we additionally prepared cryosections for CD9 and collagen type VI immunostaining and confirmed the site‐specific distribution of EVs under these native conditions.

We isolated EVs from synovial fluid and cell culture medium of articular chondrocytes and demonstrated that chondrocyte‐derived EVs are capable of exhibiting autofluorescence under the given experimental conditions. This supports previous data, which showed that EVs can produce MPAF signals (Sorrells et al. 2021, 2024; You et al. 2019). Together, these results suggest that EVs do produce at least a major portion of the extracellular MPAF signal due to the correlating distribution. On the other hand, other sources might contribute to the site‐specific MPAF signals shown here. Elastin fibres, for example, are MPAF‐positive and some of these fibres were visible in MPAF images of the superficial matrix (Figure 2, arrows with F). These fluorescent fibres did not produce a SHG signal and have been well described as elastin‐fibres by others (Mansfield et al. 2009; Boyanich et al. 2019; Yu and Urban 2010). We have confirmed that these fibres are elastin‐immunopositive and while there is no correlating elastin signals in EV‐enriched areas lateral to the cells and above the cells (which showed the MPAF signal accumulations described above). Therefore, elastin is not responsible for these MPAF signals (Figure S2). Other MPAF sources, such as collagen degradation products or metabolic byproducts of cartilage tissue components, were not further investigated in this study (Yeganegi et al. 2023).

We demonstrated the possibility of visualizing EVs by scanning intact fresh cartilage through its surface without the need of histological sampling and preparation, which would be ideal for the usage in clinically applicable endomicroscopy, which is gaining attention for potential in‐vivo diagnostics (Tschaikowsky et al. 2021; Kim et al. 2022). The ability of the EVs to produce a MPAF signal might therefore be a potential feature for tracking the path of EVs through different types of tissue and under different conditions, maybe even in‐vivo.

The OARSI score and grade were determined in order to classify the tissue samples as either healthy or slightly osteoarthritic (Pritzker et al. 2006; Little et al. 2010). The points given in the scoring system were given via scoring the worst part of each sample. This ensured that the used healthy cartilage was barely affected by degenerative alterations. By choosing bovine tissue over human tissue it was possible to harvest healthy cartilage samples, since human donor cartilage from joint replacement surgery is usually influenced by strong osteoarthritic changes with a concordant pro‐inflammatory milieu, which has been described to go hand in hand with a change in the vesicle expression of the joint fluid and the cartilage (Yang et al. 2023; Derfus et al. 1996). Therefore, the decision was made to investigate bovine tissue from the femoropatellar groove, as it has been used in previous studies and mainly represents a healthy tissue (Behrendt et al. 2018, 2016). In the distal femoropatellar groove it has been described that osteoarthritis develops regularly (Heinola et al. 2014). In three knees, we isolated cartilage from that region and found cartilage samples with OARSI grades one and two, representing the biological onset of osteoarthritis in cartilage tissue. These samples were investigated in order to study MPAF and CD9 distribution in early osteoarthritic tissue.

## 5 Conclusion

The findings of this study lay the groundwork for deeper investigations into the spatial distribution of extracellular vesicles (EVs) in cartilage biology and early osteoarthritis development. The non‐homogeneous distribution of EVs around superficial chondrocytes in healthy cartilage, which is missing in early OA tissue, suggests that these vesicles may play a highly localized role in maintaining cartilage integrity or mediating tissue responses. Future research needs to focus on understanding the functional significance of these specific topographic EV patterns, particularly in relation to cartilage regeneration, intercellular communication, and early disease mechanisms. Expanding these investigations into human cartilage and osteoarthritic models will be crucial in determining whether the observed patterns hold clinical relevance, potentially opening new avenues for diagnostic markers or therapeutic targets in osteoarthritis. Furthermore, leveraging advanced imaging techniques, such as non‐invasive multiphoton microscopy could provide a powerful tool for tracking EV dynamics in real‐time, aiding in the development of EV‐based treatments for joint degeneration and repair.

## Author Contributions

**Florian Gellhaus**: study design; acquisition and analysis of the data; writing. **Bodo Kurz**: study design, analysis of data, supervision, writing. **Jan‐Tobias Weitkamp**: supported immunohistochemical experiments. Wiebke Lückstädt: Isolation of extracellular vesicles, biochemical analysis, imaging and dynamic light scattering. **Peter Behrendt**: supported immunohistochemical experiments. **Greta Ahrens**: tissue harvesting, sample preparation and analysis. **Christine Desel**: supporting the non‐labelling imaging acquisition. **Bernd Rolauffs**: supervision, supporting the non‐labelling imaging acquisition. All authors read and approved the final manuscript.

## Ethics Statement

Inquiry revealed that no consultation with the local ethics committee was necessary. The tissue samples used were from bovine knee joints and were obtained from a local butcher. The joints were discarded during the slaughter process.

## Conflicts of Interest

The authors declare that they have no competing interests.

## Supporting information




**Supplementary Figures**: jev270183‐sup‐0001‐figureS1‐S2.docx

## Data Availability

The dataset analysed during the current study is available from the corresponding author on reasonable request.
