# Supplementary material for: Spatial Distribution of Extracellular Vesicles, Autofluorescence and CD9 Positivity Around Chondrocytes in the Superficial Layer of Articular Cartilage
Source: J Extracell Vesicles. 2025 Oct 21;14(10):e70183. doi: 10.1002/jev2.70183 (PMC12538814; doi:10.1002/jev2.70183)
Supplement: Supplementary file 1 — Supplementary Figures: jev270183‐sup‐0001‐figureS1‐S2.docx [file JEV2-14-e70183-s001.docx]

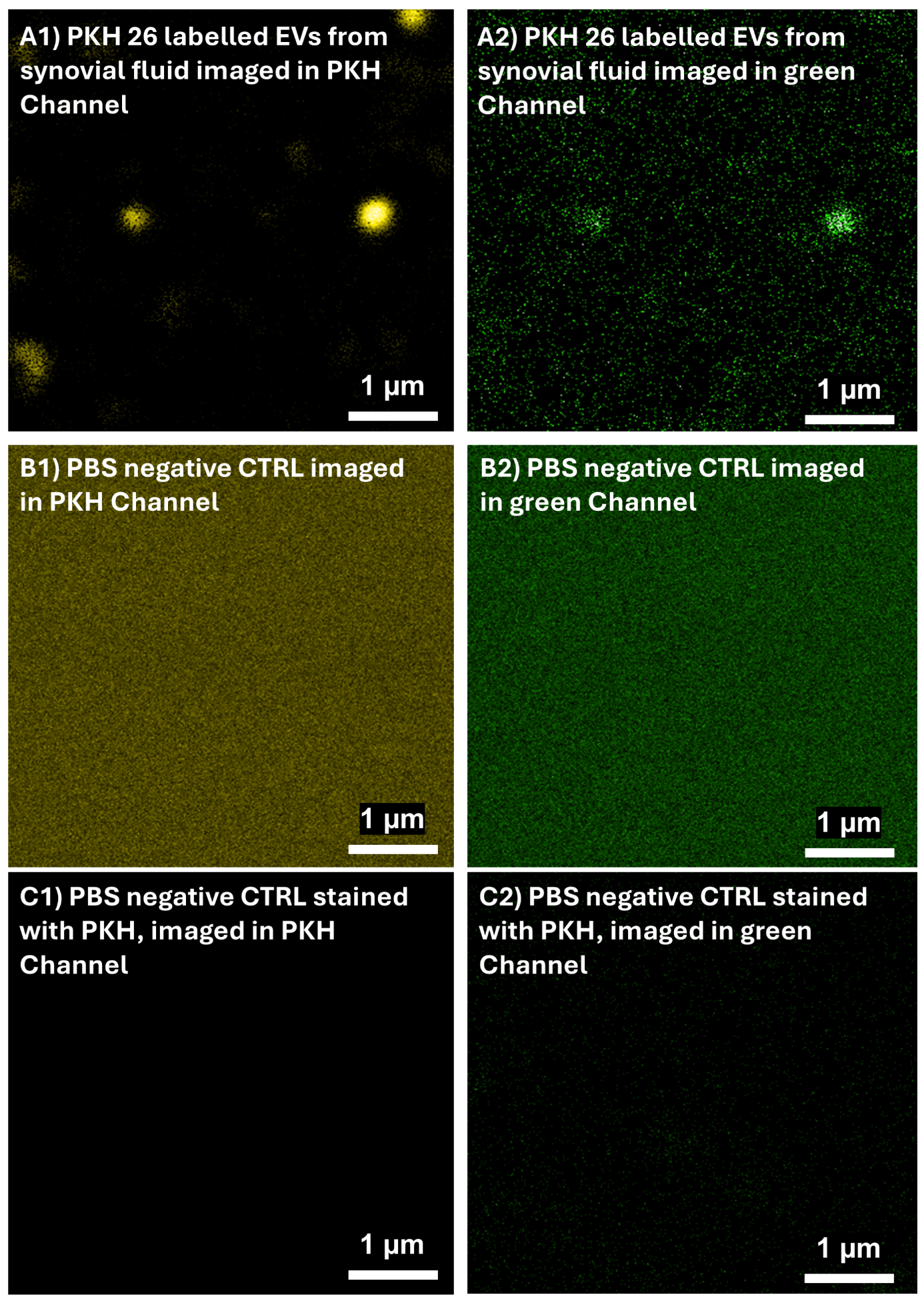


Supplemental Figure 01A: A1/A2 Colocalization of PKH 26-positive EVs with autofluorescence imaging. Cross-talking between the channels possible. B1/B2 Imaging of a PBS negative control treated as EVs being prepared for autofluorescence imaging, but with a PBS probe instead of EVs from cell culture medium or synovial fluid. C1/C2 Imaging of a PBS negative control treated as EVs being labelled with PKH 26 for imaging, but with a PBS probe instead of EVs from cell culture medium or synovial fluid.


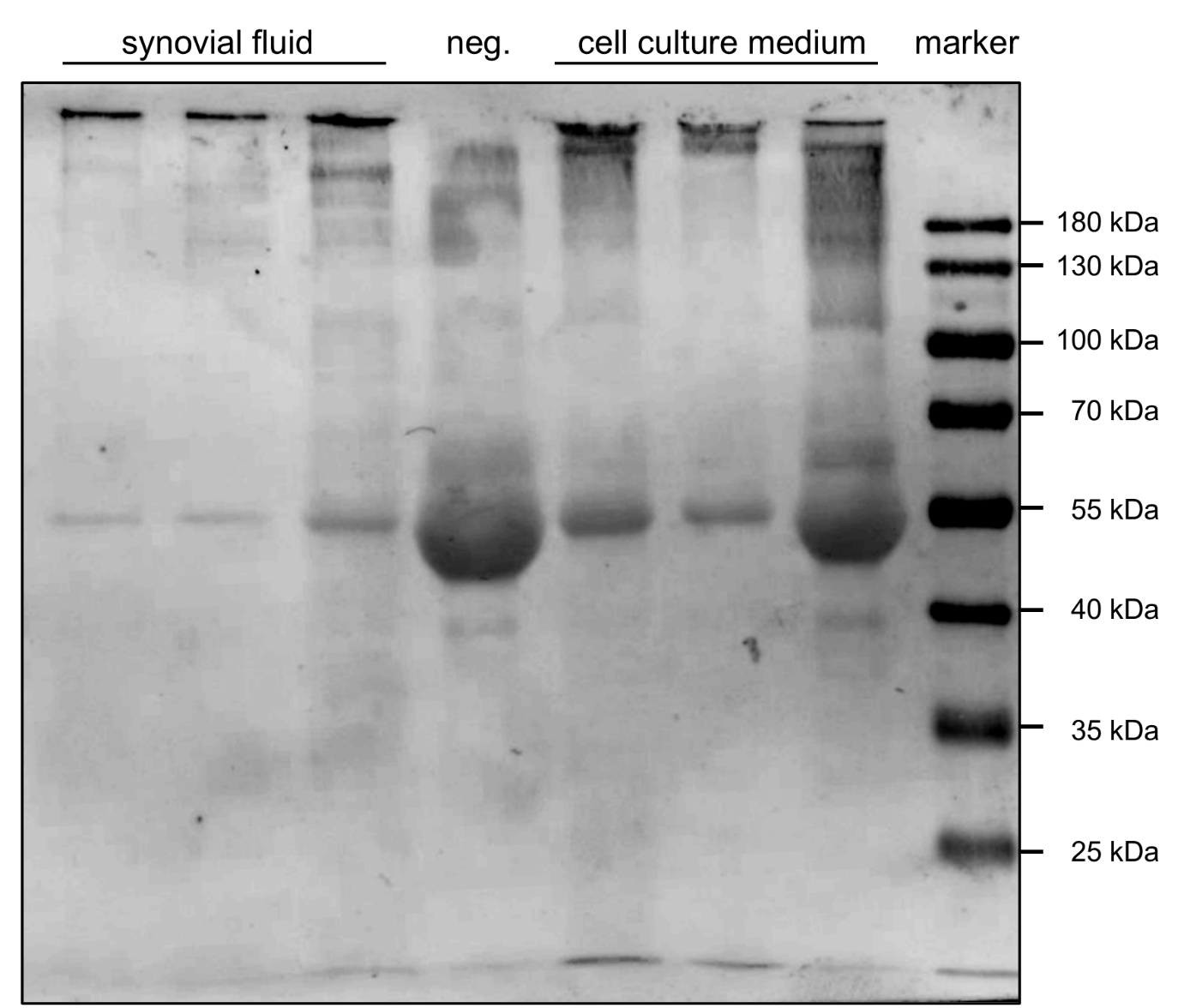


Supplemental Figure 01B: Ponceau S-stained western blot membrane shown in Figure 01 with PageRuler Prestained Protein Ladder (Thermo Fisher Scientific, Waltheim, MA, USA) to show loading control.


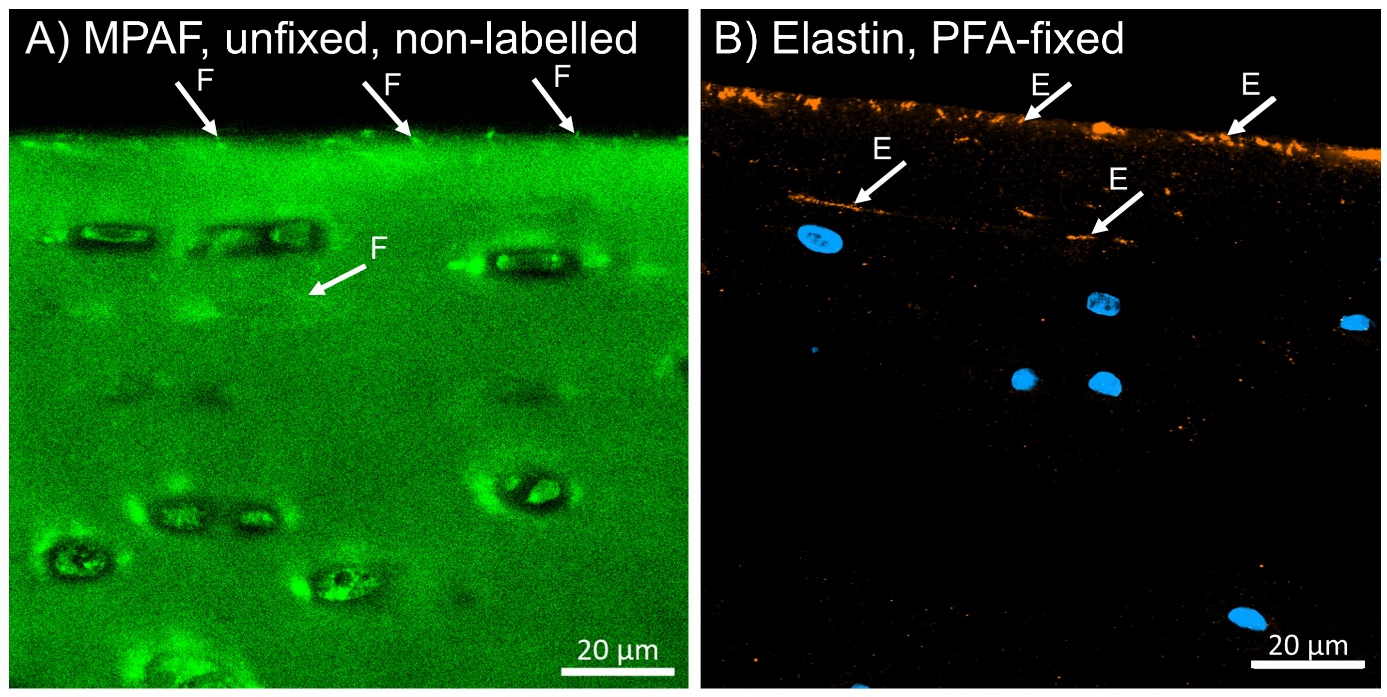


Supplemental Figure 02: Detection of elastin fibers in cartilage. In unfixed, non-labelled cartilage tissue elastin fibers produce a multiphoton-autofluorescence (MPAF) signal (arrows with “F”). Immunodetection of elastin fibers (n = 3) show the main position of elastin directly at the tissues surface, or as single fibers inside the cartilage matrix (arrows with “E”), comparable to the location found in the MPAF imaging. No elastin was detectable in the location lateral or above the cells.
